# Quantification of myocardial deformation in children by cardiovascular magnetic resonance feature tracking: determination of reference values for left ventricular strain and strain rate

**DOI:** 10.1186/s12968-016-0310-x

**Published:** 2016-12-05

**Authors:** Florian André, Daniëlle Robbers-Visser, Astrid Helling-Bakki, Angela Föll, Andreas Voss, Hugo A. Katus, Willem A. Helbing, Sebastian J. Buss, Joachim G. Eichhorn

**Affiliations:** 1 Department of Cardiology, Angiology and Pneumology, University of Heidelberg, Heidelberg, Germany; 2 Department of Pediatrics, Division of Pediatric Cardiology and Department of Radiology, Erasmus MC-Sophia Children’s Hospital, Rotterdam, The Netherlands; 3 Department of General Pediatrics, University of Heidelberg, Heidelberg, Germany; 4 Institute of Psychology, University of Heidelberg, Heidelberg, Germany; 5 Klinikum Leverkusen, Children’s Hospital, Am Gesundheitspark 11, 51375 Leverkusen, Germany

**Keywords:** Cardiovascular magnetic resonance, Feature tracking, Pediatrics, Strain, Physiology, Reference values

## Abstract

**Background:**

The objective assessment of global and regional cardiac function in children has shown to be clinically relevant but is challenging to conduct. Cardiovascular magnetic resonance (CMR) has emerged as a valuable diagnostic modality especially in patients with cardiomyopathy or congenital heart disease. However, data on the normal cardiac deformation in children assessed by CMR is lacking at present. Thus, the aim of this study was to provide reference values for cardiac strain and strain rate in children and adolescents derived from CMR feature tracking (FT) measurements.

**Methods:**

In this binational study, eighty children and adolescents (age 0.4–18.0 years, 41 male, 39 female) free from cardiac diseases from two centers underwent CMR in 1.5 T whole-body scanners in supine position. Global peak radial, circumferential and longitudinal systolic strains as well as the corresponding early peak diastolic strain rates were assessed applying FT on short axis as well as 3- and 4-chamber views of standard cine steady-state free precession images.

**Results:**

The difference between genders yielded no significance for all assessed strains. Yet, all strains showed a significant parabolic relation to age and an even stronger one to body surface area (BSA). Therefore, BSA-specific reference values were determined using a polynomial regression model. The apical cardiac segments featured significant higher peak circumferential but lower peak radial systolic strains than the midventricular and basal segments (all *p* < 0.001).

**Conclusions:**

The assessment of cardiac deformation by CMR-FT is feasible in children. This is the first CMR study providing specific reference values for FT-derived strain and strain rate in the pediatric age range.

**Electronic supplementary material:**

The online version of this article (doi:10.1186/s12968-016-0310-x) contains supplementary material, which is available to authorized users.

## Background

The objective assessment of cardiac function in children is still challenging despite the tremendous advances of the last decades [1]. Echocardiography (EC) is the standard imaging modality in pediatric cardiology due to its wide availability, but it faces several limitations especially in children with congenital heart disease (CHD) [2]. Cardiovascular magnetic resonance (CMR) has been established as an important non-invasive diagnostic modality for children and adults with CHD as it can provide anatomical and physiological information in a wide variety of ventricular geometry [3, 4]. In addition to a precise quantification of cardiac volumes and mass, CMR offers the possibility to assess the cardiac deformation measured by cardiac strain. Previous studies demonstrated that the cardiac strain is of prognostic value in adults as well as in children with cardiomyopathies [5, 6]. Recently, feature tracking (FT), which was originally developed for the cardiac deformation analysis of EC studies, has been applied to CMR steady-state free precession (SSFP) images. In contrast to other CMR strain quantification methods as MR tagging, strain-encoding (SENC) or displacement encoding with stimulated echoes (DENSE) imaging, FT does not need additional time-consuming scans or extra MR sequences. The correlation between CMR-FT measurements and EC wall deformation analysis is high [7, 8]. Likewise, the accordance with MR tagging has shown to be good [9–11] and reference values for CMR-FT derived cardiac strains in adults have been published recently [12, 13]. Yet, there is only little data on the cardiac deformation in children. Therefore, the aim of this study was to determine reference values for the left ventricular (LV) deformation in children and adolescents applying FT on standard SSFP series.

## Methods

### Study population

The study population consisted of 80 children and adolescents free from cardiac diseases who were recruited at two departments of pediatric cardiology in Germany (*n* = 27) and the Netherlands (*n* = 53). The age ranged from 0.4 to 18.0 years and both genders were represented nearly equally (41 male, 39 female). The study was approved by the local ethics committees and in accordance with the Declaration of Helsinki. The parents gave informed consent to the participation of their children. A fraction of the study population was part of a previous trial on reference values [14].

### Clinical data

Height and weight were measured and the body surface area (BSA) was calculated using the Mosteller formula (*n* = 79) [15].

### CMR Examinations

CMR examinations were performed on two different 1.5 T whole body MR scanners (Magnetom Symphony 1.5 T, Siemens Healthcare, Erlangen, Germany, and Signa 1.5 T, GE Healthcare, Chalfont St Giles, Great Britain) using a phased-array surface coil in supine position. Some of the younger probands (<6 years) needed a mild sedation. However, general anesthesia was required in none of the subjects. A vector electrocardiogram was used for R-wave triggering. Cine images of short axis views, normally covering the entire LV from base to apex, as well as of 3- and 4-chamber views were acquired using a standard SSFP sequence. Imaging parameters were TE 1.1–1.2 ms, TR 40–42 ms, flip angle 80°, field of view (FOV) 160–190 mm x 125–156 mm, matrix 256 × 256 and slice thickness 4–8 mm for the Magnetom Symphony scanner and TE 1.5 ms, TR 3.5 ms, flip angle 45°, FOV 280–370 mm, phase FOV 0.75, matrix 160 × 128 and slice thickness 7–10 mm for the Signa scanner. The number of acquired phases at the Magnetom scanner varied between 20 and 50 per cardiac cycle. Series with a low number of phases were mostly acquired in children with high heart rates. Thus, the temporal resolution was 20.3 (19.0 to 25.6) ms. At the Signa scanner, 30 phases per cardiac cycle were reconstructed consistently resulting in a temporal resolution of 24.7 (22.4 to 27.4) ms.

### LV volumetry and heart rate

Heart rate was measured during the image acquisition and retrieved from the SSFP images used for strain analysis. End-diastolic and end-systolic volumes (EDV, ESV) as well as LV mass were obtained from short axis stacks and stroke volume (SV) and ejection fraction (EF) were calculated (*n* = 77).

### FT Measurements

**Fig. 1:**
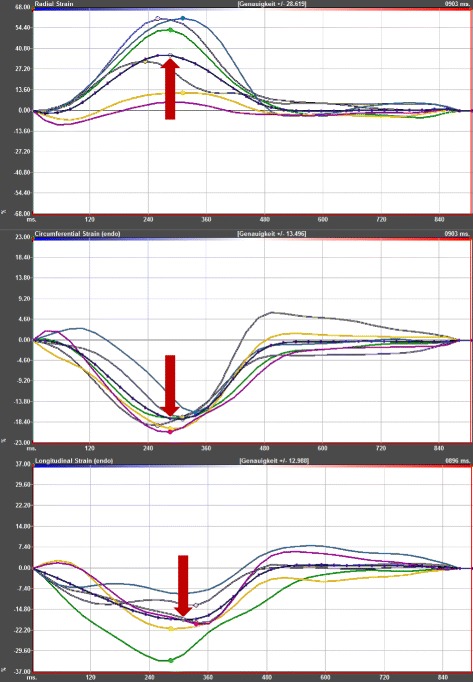
Example of a strain curve. The figure displays typical curves of the radial, circumferential endocardial and longitudinal endocardial strains. In addition to the segmental strain curves, the mean strain curves are given as dotted lines and are highlighted by red arrows. The global peak systolic strain values result from the average of the segmental peak systolic strain values. The measurements were obtained from the short and long axis acquisitions of a 16-year-old girl with a BSA of 1.34 m^2^

The strain analysis was conducted on the acquired LV short and long axis views applying the 2D CPA MR feature tracking software (TomTec Imaging Systems, Unterschleißheim, Germany). The endocardial and epicardial borders were manually set in end-diastole and the software tracked image features as signal inhomogeneities, tissue patterns of the myocardium or anatomical structures throughout the entire cardiac cycle. The movement of the features in relation to each other was employed to determine the cardiac strain. A cine mode allowed for a visual control of the tracking quality by showing the tracked endocardial and epicardial borders as well as the resulting strain curves (Fig. 1). In case of an inadequate tracking, a manual correction and re-tracking of the series could be performed. Segments not suitable for a reliable tracking were excluded from analysis. We used the anterior insertion of the right ventricle to define the LV cardiac segments according to a 16-segment model, which conforms to the standard 17-segment model of the American Heart Association omitting the apical cap [16]. As in previous studies, the segmental peak systolic strain values were derived from the average of three measurements in each patient [12]. The global peak radial and circumferential systolic strains were derived from the averaged peak segmental systolic strain values of an apical, a midventricular and a basal short axis view, thus comprising 16 cardiac segments. The global peak longitudinal systolic strains were derived from the averaged segmental peak systolic strain values of a 3- and a 4-chamber view. In order to provide values for the diastolic function, the early peak diastolic strain rates were obtained from radial, circumferential and longitudinal measurements by quantifying the first diastolic peak wave. Mathematically, strain rates are defined as the derivative of strain with respect to time. Furthermore, the maximum apical and basal endocardial rotation was quantified in short axis views. Of note, clockwise rotation results in negative values and counterclockwise rotation in positive ones. Thus, absolute values were used for analysis in this study. The results are presented with the algebraic sign of the main rotation direction and the fraction of subjects with an opposed maximum rotation direction is given.

### Intra- and interobserver reproducibility

For the assessment of the intra- and interobserver reproducibility, the global peak radial systolic strain as well as the global peak circumferential and longitudinal endocardial systolic strains were measured in 20 subjects. The intraobserver reproducibility was derived from two single measurements. For the interobserver reproducibility, the same cases were evaluated by a second reader who was blinded to the obtained results.

### Statistics

Data were analyzed using MedCalc Statistical Software version 15.8 and 16.8 (MedCalc Software bvba, Ostend, Belgium). The Tukey criterion was employed on the global strain and strain rate values for outlier detection and outside as well as far out-values were excluded from further analysis. Normal distribution was assessed by the D’Agostino-Pearson test. Correlation was evaluated by the Spearman’s coefficient of rank correlation for non-parametric data. Linear and parabolic regression models were used to assess the relationship between different variables. For the comparison of two groups the Student’s *t*-test, the Welch-test or the Mann-Whitney test were employed as applicable. The Bonferroni correction was applied when necessary. For the definition of reference values, a polynomial regression model with regard to a possible multicollinearity of the variables was applied. The coefficient of variation from duplicated measurements was employed for the assessment of the intra- and interobserver reproducibility. C﻿on﻿tinuous data are give﻿n as mean ± standard deviation and non-parametric data as median (interquartile rang﻿e). A *p*-value <0.05 was regarded as statistically significant.

## Results

### Study population

**Fig. 2:**
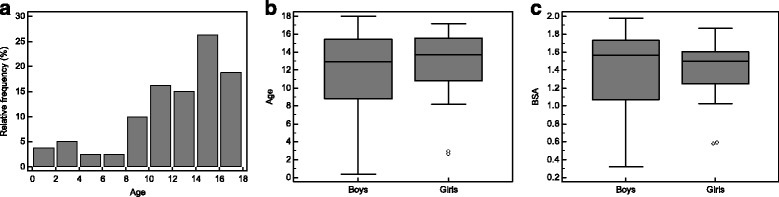
Characteristics of the study population. **a** Histogram of the age distribution of the study population. Age is given as years. **b** Age showed no significant difference between male and female subjects. Age is given as years. **c** BSA showed no significant difference between male and female subjects. BSA is given as m^2^

**Fig. 3:**
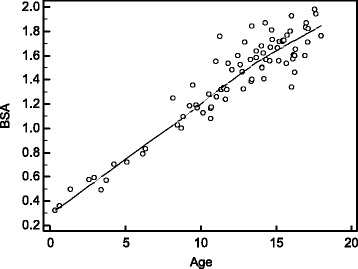
Correlation between BSA and age. BSA and age correlated strongly and highly significantly (*ρ* = 0.86, *p* < 0.001). A local regression smoothing trendline with a span of 80% is displayed. BSA is given as m^2^ and age as years

The median age of the study population was 13.4 (10.4 to 15.5) years (Fig. 2a) and the median BSA was 1.5 (1.2 to 1.7) m^2^. Age of male and female subjects was similar (13.0 (8.8 to 15.4) years vs. 13.7 (10.8 to 15.6) years, *p* > 0.4, Fig. 2b). Likewise, the BSA showed no significant difference between boys and girls (1.6 (1.1 to 1.7) m^2^ vs. 1.5 (1.2 to 1.6) m^2^, *p* > 0.6, Fig. 2c). The correlation between age and BSA was strong (ρ = 0.86, *p* < 0.001, Fig. 3). Heart rate was 84.4 ± 16.6 min^−1^ with slightly higher values in boys than in girls (88.2 ± 17.4 min^−1^ vs. 80.3 ± 14.8 min^−1^, *p* < 0.05) and a significant age-dependent decrease (R^2^ = 0.30, *p* < 0.001).

EDV (106.7 ± 36.8 ml) and SV (70.6 ± 25.1 ml) showed a significant increase with age (R^2^ = 0.64, R^2^ = 0.61, both *p* < 0.001) and an association with BSA in the parabolic regression analysis (R^2^ = 0.79, R^2^ = 0.77, both *p* < 0.001). Accordingly, ESV (34.0 (27.5 to 46.0) ml), which showed a non-parametric distribution, increased significantly with age and BSA (ρ = 0.74, ρ = 0.78, both *p* < 0.001). EF (66.0 ± 6.3%) was significantly associated with age and BSA (R^2^ = 0.12, R^2^ = 0.11, both *p* < 0.05). LV mass (82.9 ± 39.4 g) showed a significant increase with age and BSA (R^2^ = 0.50, R^2^ = 0.61, both *p* < 0.001). The differences between boys and girls were not significant with regard to EDV, ESV, SV, EF and LV mass in this study population.

### Feasibility of strain measurements

From a total number of 80 datasets consisting of short and long axis views, the analysis of the radial and circumferential systolic strains, requiring short axis views, could be performed in 74 datasets (92.5%) and the assessment of the longitudinal systolic strains, using long axis views, in 79 datasets (98.8%).

The numbers of outside values, which were identified by the Tukey criterion and excluded from further analysis, were 2 for global peak radial, 5 for global peak circumferential endocardial, 3 for global peak circumferential epicardial, 3 for global peak longitudinal endocardial and 3 for global peak longitudinal epicardial systolic strains. No far-out values were detected.

Regarding the early peak diastolic strain rates, the numbers of outside values were 3 for radial, 3 for circumferential endocardial, 0 for circumferential epicardial, 2 for longitudinal endocardial and 1 for longitudinal epicardial measurements. One far-out value of the early peak radial diastolic strain rate was excluded.

The numbers of outside values were 4 for the maximum apical endocardial and 1 for the maximum basal endocardial rotation. No far-out values were detected.

### Gender-related differences

The differences between boys and girls showed no significance for all assessed strains with global peak values of 23.3 ± 5.0% vs. 24.1 ± 5.0% (*p* > 0.4) for radial, −24.5 ± 3.3% vs. −24.5 ± 2.4% (*p* > 0.9) for circumferential endocardial, −14.6 ± 2.1% vs. −15.1 ± 1.9% (*p* > 0.3) for circumferential epicardial, −18.4 ± 3.5% vs. −18.5 ± 3.8% (*p* > 0.8) for longitudinal endocardial and −15.2 ± 2.7% vs. −15.5 ± 2.9% (*p* > 0.6) for longitudinal epicardial systolic strains.

Accordingly, the differences between the gender groups featured no significance for the early peak diastolic strain rates. Values were −2.1 ± 0.4 s^−1^ vs. −2.0 ± 0.3 s^−1^ (*p* > 0.5) for radial, 2.4 ± 0.6 s^−1^ vs. 2.3 ± 0.4 s^−1^ (*p* > 0.2) for circumferential endocardial, 1.4 ± 0.3 s^−1^ vs. 1.4 ± 0.3 s^−1^ (*p* > 0.7) for circumferential epicardial, 1.9 ± 0.4 s^−1^ vs. 1.9 ± 0.3 s^−1^ (*p* > 0.3) for longitudinal endocardial and 1.5 ± 0.3 s^−1^ vs. 1.5 ± 0.3 s^-1^ (*p* > 0.7) for longitudinal epicardial early peak diastolic strain rates.

### Age-related alterations

The regression analysis showed a significant age-dependency for all assessed strains. The parabolic regression equations featured better model fits than the linear equations with values of R^2^ = 0.18 vs. R^2^ = 0.14 for radial, R^2^ = 0.25 vs. R^2^ = 0.09 for circumferential endocardial, R^2^ = 0.27 vs. R^2^ = 0.10 for circumferential epicardial, R^2^ = 0.20 vs. R^2^ = 0.15 for longitudinal endocardial and R^2^ = 0.17 vs. R^2^ = 0.15 for longitudinal epicardial global peak systolic strains.

### BSA-related alterations

The model fit could be further improved by using BSA instead of age as the independent variable for the parabolic regression model leading to values of R^2^ = 0.22 for radial, R^2^ = 0.28 for circumferential endocardial, R^2^ = 0.30 for circumferential epicardial, R^2^ = 0.21 for longitudinal endocardial and R^2^ = 0.18 for longitudinal epicardial global peak systolic strains.

### Strain rates

Regarding the early peak diastolic strain rates, only the longitudinal endocardial and epicardial values (R^2^ = 0.15, R^2^ = 0.09, both *p* < 0.05) showed a significant age-dependency using the parabolic regression model. When the BSA was applied as independent variable, only the parabolic model of the early peak circumferential epicardial diastolic strain rate yielded a statistical significance (R^2^ = 0.09, *p* < 0.05).

### Influence of heart rate

Regarding cardiac strains, the regression analysis showed a significant influence of heart rate on the global peak longitudinal endocardial and epicardial systolic strains (R^2^ = 0.09, R^2^ = 0.08; both *p* < 0.05) as well as on the global peak circumferential epicardial systolic strain (R^2^ = 0.09; *p* < 0.05). However, applying a stepwise multivariable regression model on these strains with age, BSA and heart rate as independent variables, the heart rate did not remain in the final model.

Regarding the early peak diastolic strain rates, the parabolic regression analysis showed a significant heart rate-dependency of the radial, circumferential endocardial, circumferential epicardial, longitudinal endocardial and longitudinal epicardial values (R^2^ = 0.30, R^2^ = 0.36, R^2^ = 0.21, R^2^ = 0.14, R^2^ = 0.14; all *p* < 0.01). Applying a stepwise multivariable regression model with age, BSA and heart rate as independent variables on these early peak diastolic strain rates, heart rate as well as BSA remained in the final model for the radial as well as the circumferential endocardial and the longitudinal endocardial values. Age remained additionally in the final model for the early peak longitudinal endocardial diastolic strain rate. For the early peak circumferential epicardial and longitudinal epicardial diastolic strain rates, heart rate was the only variable remaining in the final model.

### Determination of reference values

**Fig. 4:**
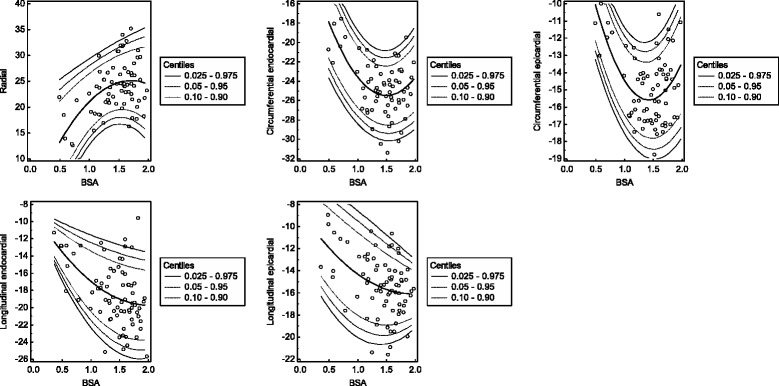
Scatter plots of the global peak systolic strains with regard to BSA. The calculated mean (central line) as well as the 0.025, 0.05, 0.10, 0.90, 0.95 and 0.975 centiles are displayed. Strain values are given as % and BSA is given as m^2^

**Fig. 5:**
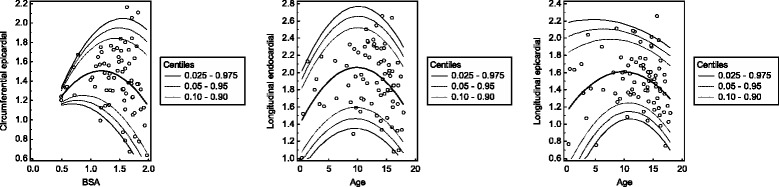
Scatter plots of the early peak diastolic strain rates with regard to BSA or age. The calculated mean (central line) as well as the 0.025, 0.05, 0.10, 0.90, 0.95 and 0.975 centiles are displayed. Strain rate values are given as s^−1^, age is given as years and BSA is given as m^2^

Because of its superior model fit in the prior analyses, BSA was used as independent variable for the global peak systolic strain values in the polynomial regression model. The results are given in Tables 1, 2, 3, 4 and 5 and displayed in Fig. 4. The reference values for the early peak diastolic strain rates are given in Tables 6, 7, 8 and 9 and shown in Fig. 5. Furthermore, references values for the global peak longitudinal systolic strains with respect to BSA and the early peak longitudinal diastolic strain rates with respect to age derived only from 4-chamber views are provided in an additional file (Additional file 1).

**Table 1:** Centiles of the global peak radial systolic strain

BSA	0.025	0.05	0.10	0.90	0.95	0.975
0.5	1.3	3.3	5.5	21.2	23.4	25.4
0.6	4.2	6.0	8.0	22.4	24.5	26.2
0.7	6.8	8.4	10.3	23.6	25.4	27.1
0.8	9.1	10.6	12.3	24.6	26.4	27.9
0.9	11.1	12.5	14.1	25.6	27.2	28.7
1.0	12.8	14.1	15.7	26.5	28.1	29.4
1.1	14.2	15.4	16.9	27.4	28.9	30.1
1.2	15.3	16.5	18.0	28.2	29.6	30.9
1.3	16.0	17.3	18.7	28.9	30.3	31.5
1.4	16.5	17.8	19.2	29.5	30.9	32.2
1.5	16.7	18.0	19.5	30.0	31.5	32.8
1.6	16.6	18.0	19.5	30.5	32.1	33.5
1.7	16.2	17.6	19.3	31.0	32.6	34.0
1.8	15.5	17.0	18.8	31.3	33.1	34.6
1.9	14.5	16.1	18.1	31.6	33.5	35.1

BSA is given as m^2^ and strain values are given as %

**Table 2:** Centiles of the global peak circumferential endocardial systolic strain

BSA	0.025	0.05	0.10	0.90	0.95	0.975
0.5	−23.7	−22.8	−21.7	−14.2	−13.1	−12.2
0.6	−24.9	−24.0	−23.0	−15.8	−14.8	−13.9
0.7	−26.0	−25.1	−24.1	−17.2	−16.2	−15.4
0.8	−26.9	−26.1	−25.1	−18.5	−17.5	−16.7
0.9	−27.7	−26.9	−26.0	−19.6	−18.6	−17.9
1.0	−28.4	−27.6	−26.7	−20.5	−19.6	−18.8
1.1	−29.0	−28.2	−27.4	−21.2	−20.3	−19.6
1.2	−29.4	−28.7	−27.8	−21.8	−20.9	−20.2
1.3	−29.8	−29.0	−28.2	−22.2	−21.3	−20.6
1.4	−30.0	−29.3	−28.4	−22.4	−21.5	−20.8
1.5	−30.1	−29.4	−28.5	−22.4	−21.6	−20.8
1.6	−30.1	−29.3	−28.5	−22.3	−21.4	−20.7
1.7	−30.0	−29.2	−28.3	−22.0	−21.1	−20.4
1.8	−29.7	−28.9	−28.0	−21.6	−20.6	−19.8
1.9	−29.3	−28.5	−27.6	−20.9	−20.0	−19.1

BSA is given as m^2^ and strain values are given as %

**Table 3:** Centiles of the global peak circumferential epicardial systolic strain

BSA	0.025	0.05	0.10	0.90	0.95	0.975
0.5	−13.4	−12.8	−12.2	−8.1	−7.5	−7.0
0.6	−14.4	−13.9	−13.3	−9.2	−8.6	−8.1
0.7	−15.4	−14.9	−14.3	−10.2	−9.7	−9.2
0.8	−16.2	−15.7	−15.1	−11.1	−10.6	−10.1
0.9	−16.9	−16.4	−15.9	−11.9	−11.3	−10.8
1.0	−17.5	−17.1	−16.5	−12.5	−11.9	−11.4
1.1	−18.1	−17.6	−17.0	−12.9	−12.3	−11.8
1.2	−18.5	−17.9	−17.4	−13.2	−12.6	−12.1
1.3	−18.8	−18.2	−17.6	−13.4	−12.8	−12.2
1.4	−18.9	−18.4	−17.8	−13.4	−12.8	−12.2
1.5	−19.0	−18.5	−17.8	−13.3	−12.6	−12.1
1.6	−19.0	−18.4	−17.7	−13.0	−12.3	−11.7
1.7	−18.8	−18.2	−17.5	−12.6	−11.9	−11.3
1.8	−18.6	−17.9	−17.2	−12.0	−11.3	−10.7
1.9	−18.2	−17.5	−16.8	−11.3	−10.6	−9.9

BSA is given as m^2^ and strain values are given as %

**Table 4:** Centiles of the global peak longitudinal endocardial systolic strain

BSA	0.025	0.05	0.10	0.90	0.95	0.975
0.4	−15.5	−15.1	−14.5	−10.8	−10.3	−9.8
0.5	−16.9	−16.4	−15.7	−11.3	−10.7	−10.2
0.6	−18.2	−17.6	−16.9	−11.8	−11.1	−10.5
0.7	−19.4	−18.7	−17.9	−12.2	−11.5	−10.8
0.8	−20.5	−19.7	−18.8	−12.7	−11.8	−11.0
0.9	−21.5	−20.6	−19.7	−13.1	−12.1	−11.3
1.0	−22.3	−21.5	−20.5	−13.4	−12.4	−11.6
1.1	−23.1	−22.2	−21.2	−13.8	−12.7	−11.8
1.2	−23.8	−22.9	−21.8	−14.1	−13.0	−12.1
1.3	−24.4	−23.5	−22.3	−14.4	−13.3	−12.3
1.4	−24.9	−23.9	−22.8	−14.7	−13.5	−12.5
1.5	−25.3	−24.3	−23.1	−14.9	−13.7	−12.7
1.6	−25.6	−24.6	−23.4	−15.1	−13.9	−12.9
1.7	−25.8	−24.8	−23.6	−15.3	−14.1	−13.1
1.8	−25.9	−24.9	−23.7	−15.5	−14.3	−13.3
1.9	−25.9	−24.9	−23.8	−15.6	−14.4	−13.4

BSA is given as m^2^ and strain values are given as %

**Table 5:** Centiles of the global peak longitudinal epicardial systolic strain

BSA	0.025	0.05	0.10	0.90	0.95	0.975
0.4	−16.6	−15.8	−14.8	−7.9	−6.9	−6.1
0.5	−17.4	−16.5	−15.5	−8.4	−7.4	−6.5
0.6	−18.1	−17.2	−16.2	−8.8	−7.8	−6.9
0.7	−18.7	−17.8	−16.7	−9.3	−8.2	−7.3
0.8	−19.2	−18.3	−17.2	−9.7	−8.7	−7.7
0.9	−19.7	−18.7	−17.7	−10.2	−9.1	−8.2
1.0	−20.0	−19.1	−18.1	−10.6	−9.5	−8.6
1.1	−20.3	−19.4	−18.4	−11.0	−9.9	−9.0
1.2	−20.5	−19.6	−18.6	−11.4	−10.3	−9.5
1.3	−20.6	−19.8	−18.8	−11.7	−10.8	−9.9
1.4	−20.7	−19.8	−18.9	−12.1	−11.2	−10.3
1.5	−20.6	−19.8	−18.9	−12.5	−11.6	−10.8
1.6	−20.5	−19.7	−18.9	−12.8	−12.0	−11.2
1.7	−20.3	−19.6	−18.8	−13.1	−12.4	−11.7
1.8	−20.0	−19.3	−18.6	−13.5	−12.7	−12.1
1.9	−19.6	−19.0	−18.4	−13.8	−13.1	−12.6

BSA is given as m^2^ and strain values are given as %

**Table 6:** Centiles of the early peak circumferential epicardial diastolic strain rate

BSA	0.025	0.05	0.10	0.90	0.95	0.975
0.5	1.2	1.2	1.2	1.3	1.3	1.4
0.6	1.2	1.2	1.2	1.4	1.5	1.5
0.7	1.2	1.2	1.2	1.5	1.6	1.6
0.8	1.2	1.2	1.3	1.6	1.7	1.7
0.9	1.1	1.2	1.3	1.7	1.7	1.8
1.0	1.1	1.2	1.2	1.7	1.8	1.9
1.1	1.1	1.1	1.2	1.8	1.9	1.9
1.2	1.0	1.1	1.2	1.8	1.9	2.0
1.3	1.0	1.1	1.2	1.8	1.9	2.0
1.4	0.9	1.0	1.1	1.8	1.9	2.0
1.5	0.9	0.9	1.1	1.8	2.0	2.0
1.6	0.8	0.9	1.0	1.8	1.9	2.0
1.7	0.7	0.8	0.9	1.8	1.9	2.0
1.8	0.6	0.7	0.8	1.8	1.9	2.0
1.9	0.5	0.6	0.7	1.7	1.8	2.0

BSA is given as m^2^ and strain rate values are given as s^−1^

**Table 7:** Centiles of the early peak longitudinal endocardial diastolic strain rate

Age	0.025	0.05	0.10	0.90	0.95	0.975
0.4	0.9	1.0	1.1	1.8	1.9	2.0
1.0	0.9	1.0	1.1	1.9	2.0	2.1
2.0	1.0	1.1	1.2	2.0	2.2	2.3
3.0	1.1	1.2	1.3	2.2	2.3	2.4
4.0	1.2	1.3	1.4	2.3	2.4	2.5
5.0	1.2	1.3	1.5	2.3	2.5	2.6
6.0	1.3	1.4	1.5	2.4	2.5	2.6
7.0	1.3	1.4	1.6	2.5	2.6	2.7
8.0	1.3	1.4	1.6	2.5	2.6	2.7
9.0	1.3	1.5	1.6	2.5	2.6	2.8
10.0	1.3	1.5	1.6	2.5	2.7	2.8
11.0	1.3	1.5	1.6	2.5	2.6	2.8
12.0	1.3	1.4	1.6	2.5	2.6	2.7
13.0	1.3	1.4	1.5	2.5	2.6	2.7
14.0	1.2	1.4	1.5	2.4	2.5	2.7
15.0	1.2	1.3	1.4	2.3	2.5	2.6
16.0	1.1	1.2	1.4	2.3	2.4	2.5
17.0	1.0	1.1	1.3	2.2	2.3	2.4
17.8	1.0	1.1	1.2	2.1	2.2	2.3

Age is given as years and strain rate values are given as s^−1^

**Table 8:** Centiles of the early peak longitudinal epicardial diastolic strain rate

Age	0.025	0.05	0.10	0.90	0.95	0.975
0.4	0.2	0.3	0.5	1.8	2.0	2.2
1.0	0.3	0.4	0.6	1.9	2.0	2.2
2.0	0.4	0.6	0.7	1.9	2.1	2.2
3.0	0.6	0.7	0.8	1.9	2.1	2.2
4.0	0.7	0.8	0.9	2.0	2.1	2.2
5.0	0.8	0.9	1.0	2.0	2.1	2.2
6.0	0.9	1.0	1.1	2.0	2.1	2.2
7.0	0.9	1.0	1.2	2.0	2.1	2.2
8.0	1.0	1.1	1.2	2.0	2.1	2.2
9.0	1.0	1.1	1.2	2.0	2.1	2.2
10.0	1.1	1.1	1.2	2.0	2.1	2.2
11.0	1.1	1.1	1.2	2.0	2.1	2.1
12.0	1.1	1.1	1.2	1.9	2.0	2.1
13.0	1.0	1.1	1.2	1.9	2.0	2.1
14.0	1.0	1.1	1.2	1.9	2.0	2.1
15.0	0.9	1.0	1.1	1.8	1.9	2.0
16.0	0.9	1.0	1.1	1.8	1.9	2.0
17.0	0.8	0.9	1.0	1.7	1.8	1.9
17.8	0.7	0.8	0.9	1.7	1.8	1.9

Age is given as years and strain rate values are given as s^−1^

**Table 9:** Centiles of early peak diastolic strain rates without significant age- or BSA-dependency

Strain rate	0.025	0.05	0.10	0.90	0.95	0.975
Radial	−2.9	−2.8	−2.5	−1.5	−1.4	−1.3
Circumferential endocardial	1.3	1.4	1.7	3.1	3.4	3.4

Strain rate values are given as s^−1^

### Regional cardiac deformation

**Fig. 6:**
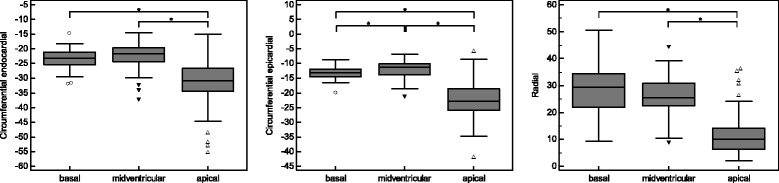
Regional cardiac deformation. Peak circumferential endocardial and epicardial systolic strains showed an increase from base to apex whereas the peak radial systolic strain decreased. *p < 0.05. Strain values are given as %

The apical cardiac segments featured significant higher peak circumferential endocardial and epicardial systolic strains than the midventricular and basal segments (apical: −30.9 (−34.4 to −26.6)% and −22.7 (−26.0 to −18.6)%; midventricular: −21.6 (−24.4 to −19.6)% and −11.3 (−13.9 to −10.1)%; basal: −23.2 (−25.4 to −21.2)% and −13.1 (−14.5 to −11.8)%; all *p* < 0.001). Yet, the peak radial systolic strain was significantly lower in the apical segments than in the midventricular and basal ones (apical: 10.1 (6.2 to 14.2)%; midventricular: 25.3 (22.4 to 30.8)%; basal: 29.3 (21.9 to 34.5)%; both *p* < 0.001). The difference between midventricular and basal segments yielded statistical significance only for the peak epicardial systolic strain (*p* < 0.001) after applying the Bonferroni correction. For a better readability, all values are given in the non-parametric notation. The results are shown in Fig. 6.

### Cardiac rotation

**Fig. 7:**
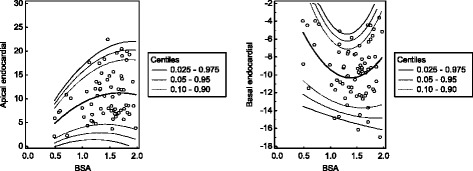
Maximum cardiac rotation. The calculated mean (*central line*) as well as the 0.025, 0.05, 0.10, 0.90, 0.95 and 0.975 centiles are displayed. In most of the subjects, the apical rotation was counterclockwise (*positive values*) and the basal rotation is clockwise (*negative values*). Of note, in 18.9% of the cases the maximum apical rotation values were negative and in 18.9% of the cases the maximum basal rotation values were positive. Rotation is given as degrees and BSA is given as m^2^

Both, maximum apical and basal endocardial rotation showed a significant parabolic association with BSA (R^2^ = 0.10, R^2^ = 0.11, both *p* < 0.05). Most of the subjects showed a counterclockwise maximum apical rotation (positive values) and a clockwise maximum basal rotation (negative values). Of note, in 18.9% of the cases the maximum apical rotation values were negative and in 18.9% of the cases the maximum basal rotation values were positive. The results are given in Tables 10 and 11 and displayed in Fig. 7.

**Table 10:** Centiles of the maximum apical endocardial rotation

BSA	0.025	0.05	0.10	0.90	0.95	0.975
0.5	0.0	0.8	1.7	8.2	9.1	9.9
0.6	0.4	1.3	2.3	9.6	10.7	11.6
0.7	0.7	1.7	2.8	11.0	12.1	13.1
0.8	1.0	2.0	3.3	12.2	13.4	14.5
0.9	1.2	2.3	3.7	13.3	14.6	15.8
1.0	1.3	2.6	4.0	14.3	15.7	17.0
1.1	1.4	2.7	4.3	15.1	16.7	18.0
1.2	1.4	2.8	4.5	15.9	17.5	18.9
1.3	1.4	2.9	4.6	16.6	18.3	19.7
1.4	1.3	2.8	4.6	17.1	18.9	20.4
1.5	1.2	2.8	4.6	17.6	19.4	21.0
1.6	0.9	2.6	4.5	17.9	19.8	21.4
1.7	0.7	2.4	4.3	18.1	20.1	21.7
1.8	0.4	2.1	4.1	18.2	20.2	21.9
1.9	0.0	1.7	3.8	18.2	20.2	22.0

Rotation is given as degrees

**Table 11:** Centiles of the maximum basal endocardial rotation

BSA	0.025	0.05	0.10	0.90	0.95	0.975
0.5	−13.5	−12.2	−10.7	−0.1	1.4	2.7
0.6	−13.8	−12.6	−11.2	−1.7	−0.3	0.8
0.7	−14.0	−12.9	−11.7	−3.1	−1.9	−0.8
0.8	−14.2	−13.2	−12.1	−4.3	−3.2	−2.2
0.9	−14.4	−13.5	−12.5	−5.3	−4.2	−3.3
1.0	−14.6	−13.8	−12.8	−6.0	−5.1	−4.2
1.1	−14.8	−14.0	−13.1	−6.6	−5.7	−4.9
1.2	−15.0	−14.2	−13.3	−7.0	−6.1	−5.3
1.3	−15.2	−14.4	−13.5	−7.1	−6.2	−5.4
1.4	−15.3	−14.5	−13.6	−7.1	−6.1	−5.3
1.5	−15.5	−14.6	−13.7	−6.8	−5.8	−5.0
1.6	−15.6	−14.7	−13.7	−6.3	−5.3	−4.4
1.7	−15.8	−14.8	−13.7	−5.6	−4.5	−3.5
1.8	−15.9	−14.8	−13.6	−4.8	−3.5	−2.4
1.9	−16.0	−14.8	−13.4	−3.7	−2.3	−1.1

Rotation is given as degrees

### Intra- and interobserver reproducibility

The intra- and interobserver coefficients of variation were 9.1 and 11.3% for radial, −0.6 and −5.2% for circumferential and −1.7 and −2.5% for longitudinal strains.

## Discussion

The quantification of cardiac deformation has emerged as a valuable method for diagnosis and risk stratification in various cardiac diseases. While reference values have been published for adults, data on the cardiac deformation in children is scarce [12, 13]. Although some previous studies had been conducted in children [17, 18], Marcus et al. were the first to publish complete reference values for radial, circumferential and longitudinal strains obtained by EC in a large cohort of healthy children and young adults [1]. Yet, normal values for CMR-derived strains in children are lacking at present. Although strain values derived from EC and CMR show a good correlation, the absolute values are not interchangeable due to methodical differences [8]. Previous studies have shown that cardiac deformation may be a prognostic parameter for different conditions, including diseased children with preserved EF [19, 20]. CMR is increasingly used clinically since it offers the possibility to assess cardiac morphology and function with high accuracy and to perform tissue characterization [21]. Therefore, the determination of CMR reference values for children is of paramount importance. By the use of FT, standard cine SSFP images can be applied for strain analysis and additional time-consuming scans, as required by MR tagging, SENC or DENSE, can be omitted. The latter point is important since the length of an examination is a relevant factor in the pediatric population [4]. FT strain analysis has shown a good correlation to MR tagging and EC wall deformation analysis in previous trials [7–11]. Furthermore, the results of studies obtained at 1.5 T and 3 T MR scanners are comparable [22]. Prior studies demonstrated that the intra- and interobserver as well as the interstudy reproducibility of FT measurements is high [12, 13], which is an important precondition for the definition of reference values.

In our study, good tracking quality could be obtained in most cardiac segments allowing reliable strain quantification. The intra- and interobserver reproducibility was adequate, which is in agreement with the aforementioned CMR trials employing FT for strain analysis in adults [12, 13].

### Influence of gender on cardiac strains

Interestingly, global peak systolic strain values did not differ significantly between male and female subjects. Previous studies establishing reference values in adult populations have shown gender-dependent differences of the cardiac deformation [10, 12, 13]. During puberty, LV mass increases particularly in boys [23]. Furthermore, cardiac contractility, which is influenced amongst others by androgens as testosterone and estrogens, changes during adolescence [24–26]. These differences between male and female cardiac morphology and physiology result in the observed gender discrepancies in cardiac deformation patterns in adults. As 75% of our study population were younger than 15.5 years, the group of adolescents was presumably too small to have a statistically significant impact. In agreement with our findings, Marcus et al. found no gender-dependent differences in their study cohort consisting of children and young adults [1].

### Influence of age and BSA on cardiac strains

All assessed global peak systolic strains showed a significant age-dependency. Interestingly, the regression model featured a non-linear alteration of the strains with age resulting in a parabolic curve shape with a maximum in puberty. In contrast to Lorch et al., who found no significant influence of age on the longitudinal strain applying linear regression models, Marcus et al. reported an age-dependency of the strains yielding a parabolic curve shape similar to our findings [1, 18]. The maximum of the strain during puberty may be ascribed to the physiological increase in cardiac contractility. As the start of puberty varies, we hypothesized that the use of anthropometric values instead of age might increase the accuracy of the regression model. And indeed, the use of BSA instead of age as independent variable in our study improved the model fit leading to a more realistic depiction of the strain alterations. Correspondingly, Kampmann et al. showed in a previous study on one-dimensional EC normal values in a large pediatric population a strong correlation between EC measurements and BSA in children except for newborns, who were not part of our study population [27]. With regard to children with hemodynamically relevant CHD or cardiomyopathy, the use of BSA- instead of age-derived centiles may allow for a more individual follow-up as these patients frequently show an impaired physical development. Nevertheless, the association between age and BSA was close in most of the cases of our study population resulting in the observed strong correlation.

In the current study, the BSA was calculated applying the Mosteller formula, which has been validated for children of different ages [15, 28]. Although showing a close correlation to the measured BSA in children in general, its precision may decrease in neonates and infants [29]. However, as the Mosteller formula is the current standard for the clinical BSA calculation in pediatrics and its application can still be recommended for newborns [30], we opted for the use of a single formula for the BSA calculation of the entire study population.

Of note, systolic strains show a wide biological variation even in healthy subjects as demonstrated in previous studies on adults and children [12, 13, 31, 32]. Thus, the variability of strain values observed in our as well as in prior study populations can only be ascribed partially to age or BSA. Other influencing factors are still not fully understood, proven or even known [31]. Especially in children, the available data are scarce and further research is needed to achieve a better understanding of the physiological changes of cardiac deformation during childhood and adolescence. Yet, as BSA was a significant independent variable of cardiac systolic strains, this study provides first specific reference values.

### Strains rates

The early peak longitudinal diastolic strain rates showed a parabolic curve with a maximum in early puberty. In a previous EC study of Bussadori et al., the global longitudinal systolic strain rates were higher in children than in adults [17]. However, a subdivision of the pediatric group by age was not conducted in this EC trial. In contrast to our findings, Lorch et al. observed in another EC study the highest values for the septal and lateral early diastolic strain rates in infancy and a decline until the age of 5 and 10 years, respectively, with a subsequent stabilization when comparing different age groups [18]. However, the linear regression analysis yielded no significant age-dependency. The use of different imaging techniques, applying tissue Doppler imaging only on the septal and lateral wall in the EC study and employing FT on CMR images in our study, as well as the different statistical approaches might impair the comparability of the results. Similar to the systolic strains, the early peak diastolic strain rates show a wide variance and are influenced by several factors. Especially the heart rate plays an important role being an independent variable for all assessed diastolic strain rates in the current study. Thus, we focused on the early peak diastolic strain rates, which might contribute to exclude a significant diastolic dysfunction. Yet, further studies assessing systolic and diastolic strain rates in children are needed.

### Patterns of cardiac deformation

Regarding the regional cardiac deformation, the peak radial systolic strain decreased significantly from base to apex whereas the peak circumferential endocardial and epicardial systolic strains featured a significant increase. Bussadori et al. found similar patterns for the circumferential strain in the aforementioned EC study on children and adults [17]. Augustine et al. observed an analog decrease of the radial strain in adults although the differences of the circumferential strain did not reach significance in this trial [10]. Analog to our findings, Venkatesh et al. found in a study on adults, who were free of cardiac diseases, an increase in the circumferential strain and a decrease in the radial strain from base to apex employing MR tagging [33].

Of note, the different systolic strains (radial, circumferential and longitudinal) are interrelated and do not directly reflect the myocardial fiber orientation. For example, the shortening and accompanying thickening of the epicardial and endocardial more longitudinal oriented fibers result in a wall thickening as the myocardium is incompressible. The contraction of the circumferential oriented myocardial fibers contributes to the thickening of the wall as well. In conclusion, the radial strain, which reflects the myocardial wall thickening, results from the contraction of myocardial fibers with different orientations. Notably, an impairment of the more longitudinal oriented endocardial fibers and the associated reduction of the longitudinal strain may cause a compensation by the remaining myocardial fibers resulting in an increase in the radial strain. Consequently, strain values need to be evaluated in relation to each other and not solely.

### Application of global strain and strain rate values

Although the segmental peak systolic strains were measured by FT, we preferred the use of the global peak systolic strain values for further analysis and the determination of reference values due to their lesser variances and higher reproducibility [12]. The significant influence of age and BSA on the strain values of the developing hearts of children impedes the determination of reliable segmental strain values in a study population of this size. Yet, in general, the spectrum of diseases in pediatric cardiology does not contain conditions that lead to strictly regional cardiac deformation abnormalities as does coronary artery disease in an adult population. Even cardiomyopathies as myocarditis, which may show a regional myocardial affection, impair the global systolic strain values significantly [34, 35]. Hence, we provide global peak systolic strain values, which might contribute to a reliable distinction between normal and impaired cardiac deformation in children.

### Limitations

Although a large pediatric study population was recruited at two centers in different countries, most of the subjects were Caucasian and the possible influence of ethnicity on cardiac strain and strain rate could not be assessed. Therefore, the results may not be fully transferable to children from other ethnicities. While the strain measurements were performed at one research institution, the assessment of LV volumes and mass were conducted at the respective centers. Our study population covered a wide age spectrum, however, the youngest subject was 4 months old. Thus, we could not assess the cardiac deformation in neonates. In addition, especially the centiles at the lower end of the age and BSA spectrum may be less precise. The longitudinal strains and strain rates were evaluated in 3- and 4- but not in 2-chamber views, which might have influenced the resulting values. In this study, basic data on cardiac rotation are provided. However, more elaborate analyses including the assessment of cardiac torsion are necessary to achieve a better understanding of the cardiac deformation in children and adolescents. As some of the younger children needed mild sedation for the CMR scans, examinations were conducted in free-breathing in these subjects potentially influencing the obtained images. As the thoracic excursion in this small subgroup of young subjects is little, general anesthesia with controlled ventilation to attain breath hold would not have been appropriate. Although diverse magnetic field strengths do not impair the comparableness of FT-derived strain values, the influence of the scanner design from different manufacturers has not been fully evaluated yet [22].

## Conclusions

This is the first study providing CMR-derived reference values for cardiac deformation in children up to 18 years of age using FT. These values may aid to the detection of an impairment of the LV function in clinical routine. As strain values alter during childhood and adolescence, this study provides specific reference values. Further studies may address the incremental benefit of strain measurements for the detection of cardiac dysfunction and the assessment of prognosis in children.

## Additional file

Additional file 1: Table S1. Centiles of the global peak longitudinal endocardial systolic strain derived only from 4-chamber views. **Table S2.** Centiles of the global peak longitudinal epicardial systolic strain derived only from 4-chamber views. **Table S3.** Centiles of the early peak longitudinal endocardial diastolic strain rate derived only from 4-chamber views. **Table S4.** Centiles of the early peak longitudinal epicardial diastolic strain rate derived only from 4-chamber views. (PDF 654 kb)

## Abbreviations

BSA: Body surface area
CHD: Congenital heart disease
CMR: Cardiovascular magnetic resonance
DENSE: Displacement encoding with stimulated echoes
EC: Echocardiography
EDV: End-diastolic volume
EF: Ejection fraction
ESV: End-systolic volume
FOW: Field of view
FT: Feature tracking
LV: Left ventricular
SENC: Strain-encoded
SSFP: Steady-state free precession
SV: Stroke volume.
