# Supplementary material for: Quantification of myocardial deformation in children by cardiovascular magnetic resonance feature tracking: determination of reference values for left ventricular strain and strain rate
Source: J Cardiovasc Magn Reson. 2016 Dec 5;19:8. doi: 10.1186/s12968-016-0310-x (PMC5248452; doi:10.1186/s12968-016-0310-x)
Supplement: Additional file 1: Table S1. — Centiles of the global peak longitudinal endocardial systolic strain derived only from 4-chamber views. Table S2. Centiles of the global peak longitudinal epicardial systolic strain derived only from 4-chamber views. Table S3. Centiles of the early peak longitudinal endocardial diastolic strain rate derived only from 4-chamber views. Table S4. Centiles of the early peak longitudinal epicardial diastolic strain rate derived only from 4-chamber views. (PDF 654 kb) [file 12968_2016_310_MOESM1_ESM.pdf]

Supplementary Table 1 – Centiles of the global peak longitudinal endocardial systolic strain derived only from 4-chamber views

BSA	0.025	0.05	0.10	0.90	0.95	0.975
**0.4**	-16.0	-15.5	-14.9	-10.4	-9.8	-9.3
**0.5**	-17.9	-17.2	-16.4	-10.9	-10.2	-9.5
**0.6**	-19.6	-18.8	-17.9	-11.4	-10.5	-9.7
**0.7**	-21.1	-20.2	-19.2	-11.9	-10.9	-10.0
**0.8**	-22.5	-21.5	-20.4	-12.4	-11.2	-10.2
**0.9**	-23.7	-22.6	-21.4	-12.8	-11.6	-10.5
**1.0**	-24.7	-23.6	-22.3	-13.2	-11.9	-10.8
**1.1**	-25.6	-24.4	-23.1	-13.6	-12.3	-11.1
**1.2**	-26.2	-25.0	-23.7	-14.0	-12.6	-11.5
**1.3**	-26.7	-25.5	-24.2	-14.4	-13.0	-11.8
**1.4**	-27.1	-25.9	-24.5	-14.7	-13.4	-12.2
**1.5**	-27.3	-26.1	-24.7	-15.1	-13.7	-12.5
**1.6**	-27.2	-26.1	-24.8	-15.4	-14.1	-12.9
**1.7**	-27.1	-26.0	-24.7	-15.7	-14.4	-13.3
**1.8**	-26.7	-25.7	-24.5	-16.0	-14.8	-13.8
**1.9**	-26.2	-25.3	-24.1	-16.3	-15.2	-14.2

BSA is given as m² and strain values are given as %

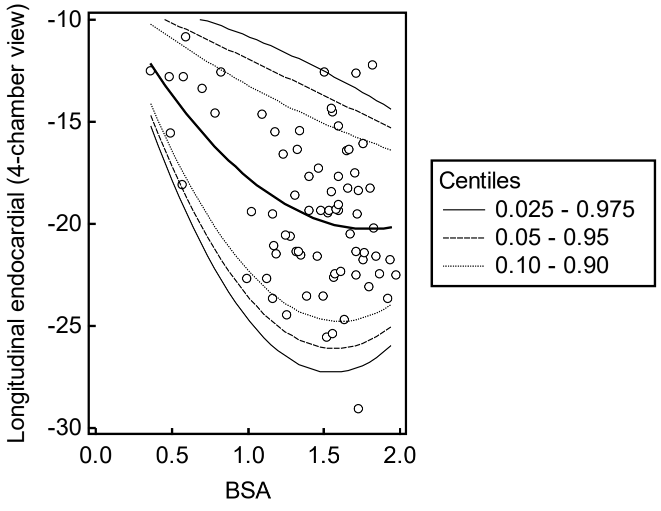

Two outside values were excluded from analysis

Supplementary Table 2 – Centiles of the global peak longitudinal epicardial systolic strain derived only from 4-chamber views

BSA	0.025	0.05	0.10	0.90	0.95	0.975
**0.4**	-17.2	-15.9	-14.5	-4.3	-2.8	-1.6
**0.5**	-17.4	-16.3	-15.0	-5.9	-4.6	-3.5
**0.6**	-17.7	-16.7	-15.5	-7.4	-6.2	-5.2
**0.7**	-17.9	-17.0	-16.0	-8.6	-7.6	-6.7
**0.8**	-18.3	-17.5	-16.5	-9.7	-8.7	-7.9
**0.9**	-18.7	-17.9	-17.0	-10.6	-9.7	-8.9
**1.0**	-19.1	-18.4	-17.5	-11.3	-10.4	-9.6
**1.1**	-19.6	-18.8	-18.0	-11.8	-10.9	-10.2
**1.2**	-20.1	-19.3	-18.5	-12.1	-11.3	-10.5
**1.3**	-20.7	-19.9	-18.9	-12.3	-11.4	-10.5
**1.4**	-21.3	-20.4	-19.4	-12.3	-11.3	-10.4
**1.5**	-22.0	-21.0	-19.9	-12.1	-10.9	-10.0
**1.6**	-22.7	-21.6	-20.4	-11.7	-10.4	-9.3
**1.7**	-23.4	-22.2	-20.9	-11.1	-9.7	-8.5
**1.8**	-24.3	-22.9	-21.3	-10.3	-8.7	-7.4
**1.9**	-25.1	-23.6	-21.8	-9.3	-7.6	-6.0

BSA is given as m² and strain values are given as %

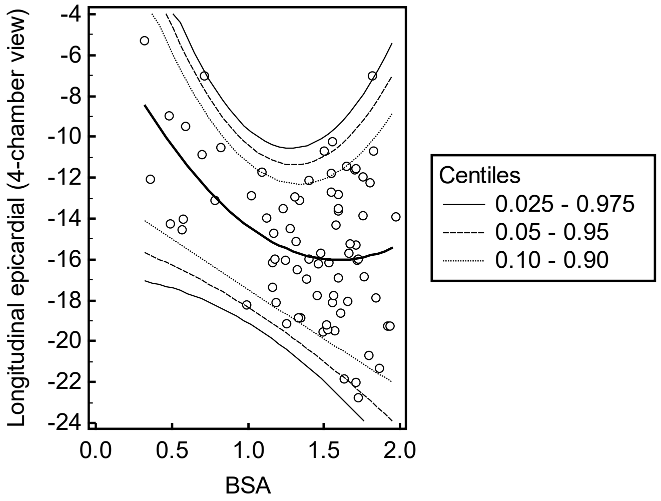

No outside values were detected

Supplementary Table 3 – Centiles of the early peak longitudinal endocardial diastolic strain rate derived only from 4-chamber views

Age	0.025	0.05	0.10	0.90	0.95	0.975
**0.4**	0.2	0.3	0.5	2.0	2.2	2.4
**1.0**	0.3	0.5	0.7	2.1	2.3	2.5
**2.0**	0.5	0.6	0.8	2.2	2.4	2.6
**3.0**	0.6	0.8	1.0	2.4	2.5	2.7
**4.0**	0.8	0.9	1.1	2.5	2.6	2.8
**5.0**	0.9	1.1	1.3	2.5	2.7	2.9
**6.0**	1.0	1.2	1.4	2.6	2.8	3.0
**7.0**	1.1	1.3	1.4	2.7	2.8	3.0
**8.0**	1.2	1.3	1.5	2.7	2.9	3.0
**9.0**	1.2	1.4	1.5	2.7	2.9	3.0
**10.0**	1.2	1.4	1.6	2.7	2.9	3.0
**11.0**	1.2	1.4	1.6	2.7	2.9	3.0
**12.0**	1.2	1.4	1.5	2.7	2.8	3.0
**13.0**	1.2	1.3	1.5	2.6	2.8	2.9
**14.0**	1.1	1.3	1.4	2.6	2.7	2.9
**15.0**	1.1	1.2	1.4	2.5	2.7	2.8
**16.0**	1.0	1.1	1.3	2.4	2.6	2.7
**17.0**	0.9	1.0	1.2	2.3	2.4	2.6
**17.8**	0.7	0.9	1.0	2.2	2.3	2.5

Age is given as years and strain rate values are given as s^{-1}

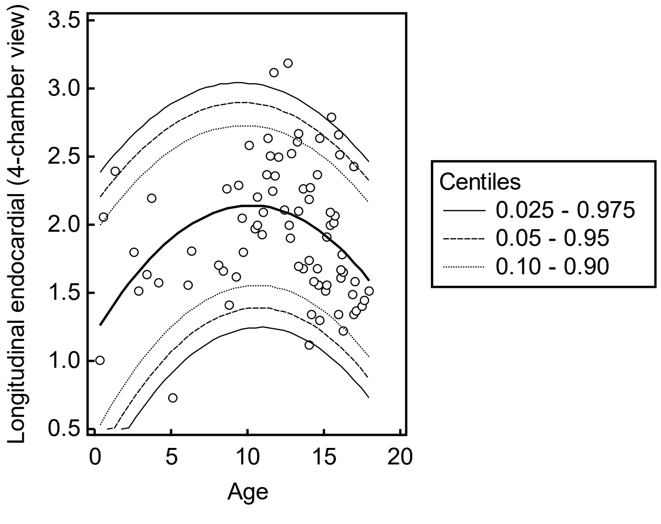

No outside values were detected

Supplementary Table 4 – Centiles of the early peak longitudinal epicardial diastolic strain rate derived only from 4-chamber views

Age	0.025	0.05	0.10	0.90	0.95	0.975
**0.4**	-0.2	0.0	0.2	2.0	2.2	2.5
**1.0**	-0.1	0.1	0.4	2.0	2.2	2.4
**2.0**	0.1	0.3	0.5	2.0	2.2	2.4
**3.0**	0.3	0.5	0.7	2.0	2.2	2.4
**4.0**	0.5	0.7	0.8	2.0	2.2	2.4
**5.0**	0.7	0.8	1.0	2.0	2.2	2.3
**6.0**	0.8	0.9	1.1	2.0	2.2	2.3
**7.0**	0.9	1.0	1.1	2.0	2.2	2.3
**8.0**	1.0	1.1	1.2	2.0	2.1	2.2
**9.0**	1.0	1.1	1.2	2.0	2.1	2.2
**10.0**	1.1	1.2	1.3	2.0	2.1	2.2
**11.0**	1.1	1.2	1.3	2.0	2.1	2.2
**12.0**	1.1	1.2	1.3	1.9	2.0	2.1
**13.0**	1.0	1.1	1.2	1.9	2.0	2.1
**14.0**	1.0	1.1	1.2	1.9	2.0	2.1
**15.0**	0.9	1.0	1.1	1.8	2.0	2.0
**16.0**	0.8	0.9	1.0	1.8	1.9	2.0
**17.0**	0.6	0.7	0.9	1.8	1.9	2.0
**17.8**	0.5	0.6	0.8	1.7	1.9	2.0

Age is given as years and strain rate values are given as s^{-1}

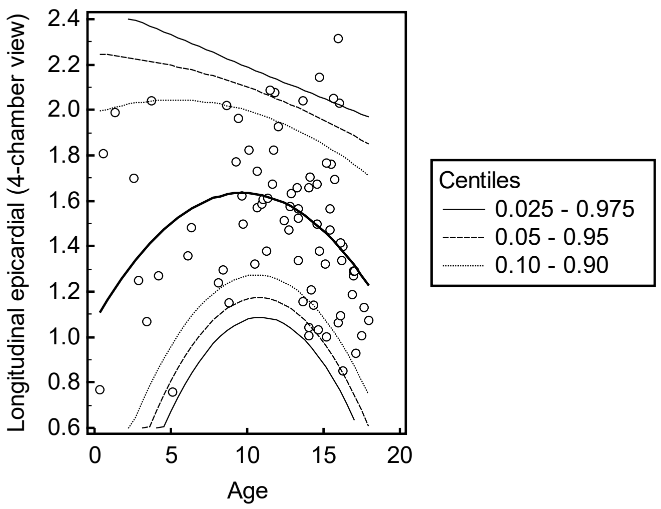

One outside value was excluded from analysis. Please note, that due to the low number of cases for young children predictions are quite imprecise as evident from the large separation of the outer centiles.
